# Legitimate suffering: a case of belonging and sickle cell trait in Brazil

**DOI:** 10.1057/s41292-021-00250-8

**Published:** 2021-10-27

**Authors:** Melissa Creary

**Affiliations:** 1 Health Management and Policy, School of Public Health, University of Michigan, 1415 Washington Heights, M3126 SPH II, Ann Arbor, MI 48109-2029 USA; 2 Office of Public Health Initiatives, American Thrombosis and Hemostasis Network, Rochester, NY USA

**Keywords:** Patient activism, Brazil, Sickle cell disease, Legitimacy

## Abstract

Patient activism organizations are formed around and seek legitimacy via both biological and biographical identities (Fassin, in: Theory Cult Soc 26(5):44–60, 2009). In the case of sickle cell disease (SCD) in Brazil, two different modes of suffering authenticate the lived experience—one is based on the disease state, the other is based on the ways in which racial inequalities and disadvantage contribute to its own suffering while also entangled with disease-based suffering. SCD is a rare genetic disorder that affects red blood cells and whose hallmark symptom is pain. This paper places an ethnographic focus on the failed mobilization of suffering by an organization leader in attempts to make claims for inclusion. The leader’s social and biological identities of mother, sickle cell trait carrier, middle class, and *mulata* disrupted biosocial cohesion. This disruption reveals a hierarchy of suffering, where some indices of suffering are delegitimized. This hierarchy illuminates how exclusion and representation work within a patient organization whose membership embody both physical and social distress.

## Introduction

Patient activist groups deploy a range of social and political identities to legitimize both themselves and their respective disease state (Rapp and Ginsburg 2001, Gibbon and Novas 2008, Hay 2010, Guell 2011, Li and Wang 2020). Brazil in particular has seen an increase in the judicialization of health since the 1990s (Nunn et al. 2009, Biehl and Petryna 2011, Biehl 2013). The 1988 Constitution, as explained below, not only gave Brazilian citizens the right to health and healthcare but also allowed them to seek legal recourse to attain these rights through the Brazilian courts. As Aureliano and Gibbons (2020) and others point out, Brazilian patient organizations often focus on how to make the state fulfill their obligation to care for them, in contrast to many Euro-American patient groups, which mobilize around finding a cure (Rabeharisoa et al. 2014). Though this activism often translates to access to innovative and expensive drugs, legitimacy extends beyond this traditional form of biolegitimacy, especially in the case of sickle cell disease (SCD) (Fassin 2009). Aureliano and Gibbons (2020, p. 249) interpret Fassin’s biolegitimacy as “humanitarian ethos…where rights are increasingly legitimized in terms of the ‘suffering body.’” Fassin (2009, p. 49) attaches biolegitimacy with “bio-inequalities…linking the biological with the biographical.” Indeed, this linking makes SCD an even more complex case. As Mota et al. (2017, p. 287) and her colleagues suggest, “In the Brazilian context, where people with sickle cell disorder are predominately Blacks, it is worth analyzing how social disadvantages can add an extra burden to the disease experience.” The claim to legitimacy by those living with SCD is via the suffering imposed on individuals and collectives by the disease, but also the suffering imposed on these same groups by virtue of their Blackness and the ways in which racial inequalities are embodied and entangled with disease-based suffering. Though disease-based suffering may allow them to gain access to certain rights under the tenets of judicialization, race-based suffering may vary in the effectiveness in which rights (read: resources) are doled out. Just as these biosocial groups use different tactics based in identity politics to navigate access to power, so too do individuals within the space of patient advocacy.

Scholars in anthropology, public health, and other fields have used the concept of biosociality to understand how biologically defined patient advocacy groups have emerged and the ways these groups become engaged with science via knowledge production and policymaking. This literature chronicles how patients, parents, and laypersons help raise funds for biomedical research, facilitate collection of blood and tissue samples, influence legislation about biomedical research, and collect and share experiences with and data about the disease (Gibbon and Novas 2008; Guell 2011; Burke 2015; Fritsch 2016). For the purposes of this paper, the concept of biosociality can help us think through how patient organizations form—usually through an adhesion of identities to serve a larger population in attaining better access to research, advanced care, and more resources (e.g., medications or infrastructure). Rabinow (1992) envisioned groups forming around biological identities marked by ill health or illness susceptibility. When civil societies form around a biological condition, they become inherently biosocial (Hacking 2006). Since its origin, biosociality has been framed through patient advocacy for technological and scientific advancement, usually with considerable attention to genetic conditions. Using the Human Genome Project as his site of inquiry, Rabinow foresaw an increasing role of genetics in the ways individuals and collectives negotiated their social experiences of illness. In Rio de Janeiro, the Rio Sickle Cell Network (pseudonym) (RSCN) seeks to influence state-wide policies for the disease, provide services and education, partner with pharmaceutical companies, and facilitate newborn screening for infants born with SCD. In this sense, RSCN demonstrates the well-established form of biosociality—a group of individuals who have formed a collective around a genetic condition. These individuals are mobilized around their disease status as a way to express their suffering and draw upon the suffering associated with racial inequality. However, a fissure occurs in their collective cohesion around shared understandings of suffering. In fact, the fissure is personified by the organization’s leader: Mira (the names of narrators and institutions are pseudonyms), a petite, fair complected woman of means who has sickle cell trait (SCT) and is the mother of two sons who have passed away from complications with SCD. This fissure raises questions about how inclusion and exclusion operate within the microcosm of a patient organization fighting for its own collective inclusion within the Brazilian society. In the case of RSCN, biosocial adhesion aligns with suffering associated with the homogenous allele distribution of SCD (HbSS) as well as the suffering associated with the poverty and social vulnerability also associated with SCD (Mota et al. 2017). The workings of the RSCN, and specifically the fissure represented by its leader, I suggest, reveal a hierarchy of suffering, where some indices of suffering are delegitimized. This hierarchy illuminates how exclusion and representation work within a patient organization formed around both biological and biographical identities.

This paper places an ethnographic focus on the failed mobilization of biological and biographical identities to make claims of inclusion in RSCN. As part of a larger study of SCD policy development, I interviewed and observed individuals and collectives associated with the SCD movement throughout Brazil. I explored the mechanisms of policy development and gained access to documents at the Ministry of Health, the Pan-American Health Organization, and other associated entities involved within the Sistema Único de Saúde or Unified Health System—the public health system for Brazil. My goals for the larger project were to investigate the simultaneous constructions of science and race through the lens of SCD across the country—allowing for additional regional constructions to make biology further malleable. To help achieve these goals, I shadowed the national coordinator for the Ministry of Health’s Sickle Cell Program in Rio de Janeiro for five months between 2013 and 2014, observing her and her everyday interactions with government officials, community leaders, patients, friends, and family members. I traveled with her to HemoRio, the blood bank and hospital where people living with SCD could get reliable care. Through this interaction, I was introduced to Mira, the president of RSCN. Located in the center of the city, within HemoRio, RSCN carries out activities to alleviate the socioeconomic situation of patients with sickle cell anemia, such as the distribution of basic food baskets and the dissemination of information about the disease to the general population, as well as legal advice. Mira is a steadfast leader who has dedicated her later years in life to running RSCN. As current president, she is well known throughout the state and nationally within the SCD movement, but her right to *be*—to be a representative for SCD and to be included in the discourse of SCD suffering—has been called to question in the past.

My methodological interest was in the discursive spaces where activism is contested and negotiated, from mundane staff meetings to congressional hearings. How might inclusion claims be performed? How are suffering and worthiness conflated? What are the ways that discourses of suffering help propel attention for a disease, for individuals, for collectives? Suffering in the sickle cell sense could also be translated into discourses around disability. The hallmark feature of SCD is pain, which connotes a visceral suffering endured by those with the genetic mutation. Suffering takes place at the population level as well in SCD’s connection to the legacies of slavery: racism, discrimination, poverty, and lack of equitable opportunities, as described further below. My goal here is not to fully explicate the rich anthropological works on suffering. Instead, I am more interested in how suffering may help us interpret authenticity claims—what kinds of suffering are allowable and acceptable for inclusion into SCD patient activism spaces?

## Framing sickle cell disease in Brazil

SCD has been a site of interrogation across disciplines, ranging from basic science (microbiology, immunology, biochemistry) to social sciences and humanities (sociology, anthropology, psychology, history, and ethics). On the cellular or molecular level, SCD remains controlled. Studies from this realm tell us that it is a genetic blood disorder of hemoglobin that damages and deforms red blood cells. The sickle-shaped red cells sometime break down (hemolysis) and cause anemia. They also obstruct blood vessels, causing ischemic organ damage and episodes of unpredictable, recurrent, and sometimes severe pain in those who suffer from SCD. There are several common variants of SCD: homozygous SS or two sickle alleles, the most common and severe form of the disease (inheritance of one sickle cell gene from each parent); heterozygous SC, a milder form of the disease (inheritance of one sickle cell allele and one allele for another abnormal type of hemoglobin called “C”); and S-beta-thalassemia (inheritance of one sickle cell allele and one allele for beta-thalassemia, another inherited hemoglobinopathy). Individuals who inherit the sickle cell gene from one parent but whose other copy of the gene is normal are healthy carriers of the disorder and are said to have sickle cell trait (SCT) (Dauphin-McKenzie et al. 2006). Sickle cell syndromes (hemoglobin variant groupings) occur in higher frequency in people from geographic areas where malaria is, or was, endemic. Those with SCT have protection against severe malaria infection (Platt and Sacerdote 2002).

Individuals with SCD are at risk for unexpected, intermittent, and, at times, life-threatening complications, including pain, infection, stroke, joint necrosis, and/or major organ damage, as well as psychosocial adjustment issues. SCD accounts for a notable proportion of visits to emergency departments and admissions to general pediatric and hematology units. One of the most common problems experienced with SCD is the pain associated with unpredictable vaso-occlusive crises or episodes. Vaso-occlusive crises are the clinical hallmarks of SCD. These events result in recurrent painful episodes, and both acute and chronic pain are associated with SCD in adults and children (Stinson and Naser 2003). SCD is often associated with chronic suffering, distress, neglect, and stigma. These descriptors are often intertwined with the life experiences of some of the narrators in my study outside of their illness narrative (Rouse 2009; Elander et al. 2011; Royal et al. 2011).

When we zoom out from the cellular level to the individual- and population-based level—taking the study of the racialized disease outside the lab—cultural, social, and political patterns problematize the once static framing of the disease. Race is made multiple, as M’Charek (2013) suggests. Tapper (1999) has described how cellular sickling was associated with African bodies by colonial medical doctors, whose data were later used by medical researchers in the United States to conclude that SCD was a ‘Black’ disease based on assumed genetic continuity between Africans and US Blacks. In the 1940s in Brazil, miscegenation was the hallmark issue of SCD studies. Dominant discourses during this period promoted whitening as a solution for Brazil’s ‘racial problem’; according to these theories, racial mixture could help eliminate SCD (Cavalcanti and Chor Maio 2011). It was thought that SCD was on the decline in Brazil during this time because of miscegenation and that miscegenation actually aided in the elimination of pathology. This biological association with Blackness would later serve as a platform for the creation of specific SCD-based policies in Brazil (Creary 2018). The social construction of SCD and SCT and its ties to nationality, race or skin color, ancestry, geographic location, social class, and various forms of capital force us to move from the cellular environment to the societal environment in which bodies with SCD exist.

SCD is one of the most prevalent hereditary diseases in Brazil. Though there is not reliable national data on prevalence, it is widely recognized that the Northeastern Region of the country has more cases of SCD than other regions. For instance, the estimated prevalence in Bahia is about one out of 600 persons; in Rio de Janeiro, one out of 1200; and in Rio Grande do Sul, about one out of 10,000. It is estimated that between 1 and 6 percent of the Black population have the sickle cell trait (Monteiro and Silva 2017). Thus, SCD is considered an important public health problem, given the number of estimated people with trait and disease, as well as its associated high mortality rate (Brasil Ministerio da Saude 2001). The lack of medical services, regular follow-up, and genetic counseling, and subsequent dependency on the health system resulted in calls for a comprehensive sickle cell program in Brazil (de Paiva et al. 1993; Brasil Ministerio da Saude 2001; Kikuchi 2007; Guimarães et al. 2009, Fernandes et al. 2010).

After many years of grassroots activist work and an eventual increase in political will with political regime changes, the Política Nacional de Saúde Integral da População Negra, or National Health Policy for the Black Population (NHPBP), was drafted in 2006 and approved in 2009. The policy—lauded as a successful achievement in the name of judicialization (Gibbon and Aureliano 2018)—was created to address health disparities and inequities that were shown to exist within the Black population. The policy encouraged, if not required, that civil society be involved in governmental relations and policymaking. Much as SCD was chosen as a “flag to demand health rights specific to the Black race” in the development of the NPHBP (Creary 2018, p. 124), so too did the civil society associations turn towards SCD and, by proxy, Blackness to denote a particular kind of suffering that constituents could rally behind.

SCD is often associated with suffering. From the perspectives of compassionate hematologists, to the language often found in fundraising materials and even official policy text, the narratives of suffering are often strategically deployed by individuals and collectives to garner political will, empathy and understanding, and material resources to benefit the SCD community. Rouse (2009, p. 137) understands suffering to be an aggregate of personal experiences and culture—in that culture determines disease symptomology. “While collective representation of sickle cell suffering—shared historical memory, disease/treatment paradigms, and political objectives—have been used to gain political advantage and increase access to resources and power, these discourses are clearly tied to their objective” (Rouse 2009, p. 137). In a study that assessed Black SCD patients’ beliefs and attitudes around race, ancestry, and nationality, Royal et al. (2011) found that with regard to the effect of race, some participants expressed an increased ability to cope via shared experience with other Black patients. Consider these remarks from one of the study’s respondents: “Being Black and knowing that most people that suffer from SCD are Black also makes it a little easier to deal with because I can associate with more people suffering with the disease” (Royal et al. 2011, p. 398). As Fullwiley (2006) suggests in her case of SCT in Senegal, a biosociality of suffering is enacted as effectively as the formation of community around genotype.

In the United States, the 1970s transformed the political presence and pertinence of SCD. SCD proved to be an effective vehicle for the Black Panther Party’s political ideology. As a condition of blood, SCD evoked kinship and common autochthony. This bond entitled the Party to speak to and for the experiences of Black suffering and to ground these claims in the history of the African diaspora (Nelson 2011). This suffering was not just associated with health. The adult population of people living with SCD are often characterized as coming from lower-middle-class and poor families, heavy utilizers of Medicaid and other public assistance, less educated, underemployed, and associated with drug use and abuse. In the case of Brazil, these characteristics ring true, but are compounded by the phenotypic spectrum.

## Phenotypic paradigms

While considered a monogenic disease, SCD presents with polygenic phenotype (Driss et al. 2009; Ballas et al. 2010; Fullwiley 2010). The phenotype of skin color signifies race for some, especially so for some in Brazil. Unlike US conceptions of race, which rely on the idea of the one-drop rule or hypodescent, Brazilians often use color (*côr*) terms rather than racial ones to describe themselves. We must recognize that different actors utilize different racial classification systems. Since 1991, the Brazilian census has employed the categories white (*Branco*), brown (*Pardo*), black (*Preto*), Asian (*Amarelo*), and Indigenous *(Indígena*). *Pardo* captures almost everyone in the continuum between *Branco* and *Preto*, though it is important to note that it goes beyond the admixture of white and Black, and includes other categories (i.e., admixture of Black and Indigenous). Created by the state and shifting significantly over the course of the nineteenth and twentieth centuries, these terms are the formalized categories used to measure race in early twenty-first-century Brazil (Nobles 2000; Telles 2004).

In addition to the formal system, there are a multitude of informal terms and categories; Brazilians describe themselves with many different monikers. Schwarcz (2003) lists 136 different self-ascribed *côr* definitions from a survey administered by the Instituto Brasileiro de Geografia e Estatística. *Côr* refers to both phenotype (skin color, hair type, nose shape, lip shape) and social position (Nobles 2000; Parra et al. 2003; Telles 2004; Travassos and Williams 2004). This collection of data suggests that the notion of Brazilian racial fluidity is prominent even in state-produced documents. Hordge-Freeman (2013) highlights the applicability of the concept of a phenotypic continuum to describe the racial structure of Brazil, with Blackness at one end of the spectrum and whiteness at the other. The term *côr* encapsulates the importance and hierarchy of phenotype for some Brazilians, many of whom eschew the term *raça* (race). Racial hierarchies often rank highest the phenotypes that most closely resemble whiteness. In some cases, some phenotypic characteristics, such as hair type, carry more importance than skin color for determining *côr* (i.e., a wavy pattern of hair on a brown and dark-skinned woman may place her closer to whiteness on the continuum) (Gilliam and Gilliam 1999; Hordge-Freeman 2013).

Given the markers of suffering and the distinctness of skin color and how it operates as a lever for social mobility, it is important to examine what an ‘authentic’ person living with SCD is imagined to be—particularly by the group living with the disease. Consider Flaviola, a thirty-year-old who has characterized her life course through the lens of suffering by proxy of her Blackness:

In adolescence, there were always those girls in school who would say mean things to us, *cabelo duro* (hard or rough hair), and so forth. In adolescence, it was much harder for me because I had a lot of friends but they were all light-skinned, with long hair. And I was always the little sick girl, with courser hair. Thank God they launched *Escova Progressiva* [a hair straightening system]!

**Fig. 1:**
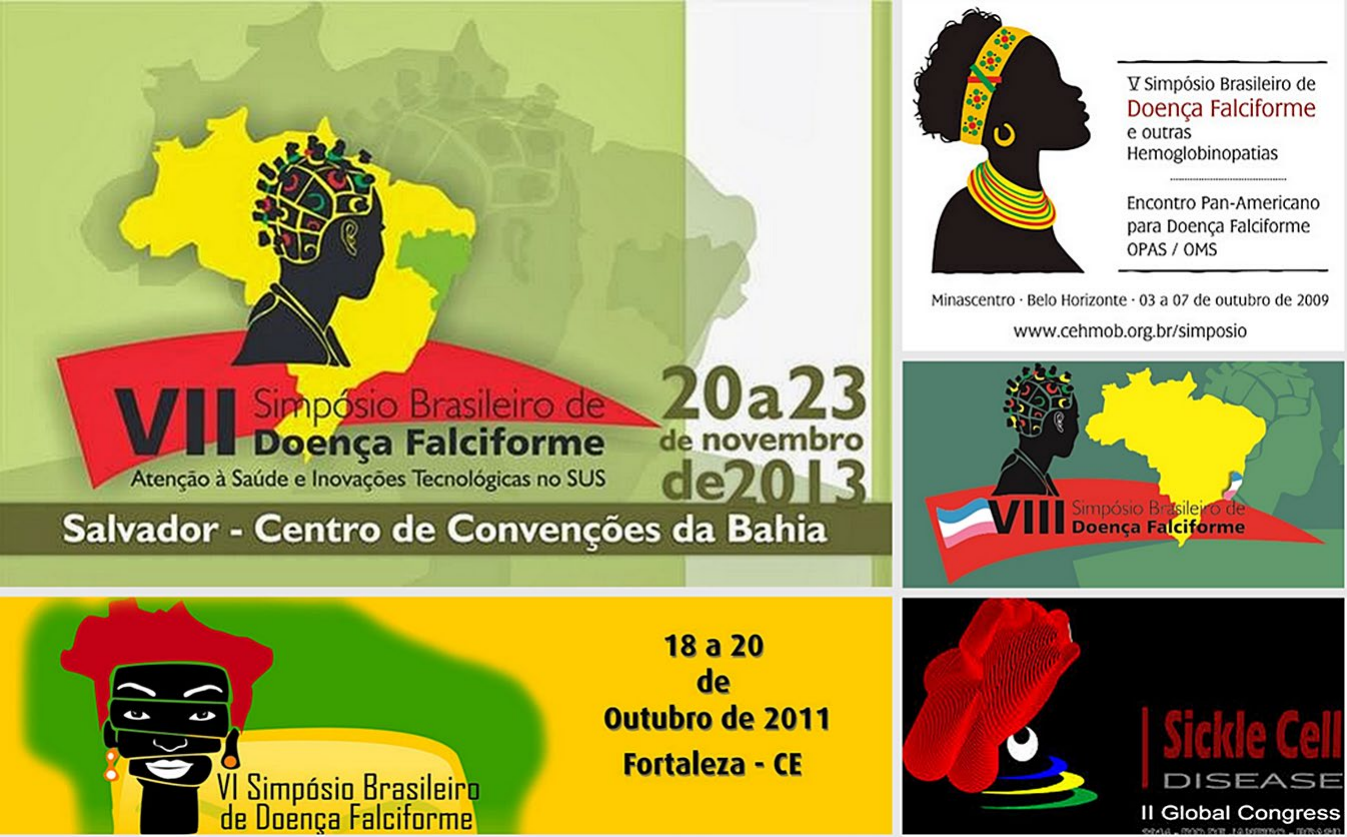
A compilation of images associated with SCD conferences in Brazil, 2011–2014

Flaviola is not the only one of my actors living with SCD who associates life hardship with their race or skin color. Many of the study respondents recount incidences of personally mediated, internal, and institutional racism. Dressler (1990) and others have discussed the impact of skin color on the instances of racial discrimination and societal inequities (Lovell and Wood 1998; Krieger 1999; Espino and Franz 2002; Telles 2004; Borrell et al. 2006; Goldsmith et al. 2006a, b; Hunter 2007; Telles 2014a). Although I spoke with persons living with SCD of all hues, the visual representation of SCD by the state and other entities in Brazil is primarily that of a dark-skinned Black body (Fry 2005). From 2009 to 2013, the state-sponsored symposium’s logo consisted of a woman’s head, distinctly adorned with African garb or accessories and shaded jet black. Between 2011 and 2013, the continent of Africa was associated with the logo (see Fig. 1). Authenticity, then, is in part often measured by skin color in the case of suffering and SCD.

For Brazil in particular, the rise of affirmative action education and health-related policies has often brought into question who has the right to make claims to the benefits bestowed to certain citizens by the government based on race or color. The politics of biological recognition depends on a ‘Black biology’ that can be specifically defined and visibly identified. For SCD in Brazil, it is not easy to disentangle biology from culture (Creary 2018); however, authenticity for the disease aligns with identity markers such as Blackness—specifically dark-skin Blackness, lower socioeconomic status, disability, and a specific type of suffering that has been catalyzed by both the biological nature of SCD as well as the societal nature of disadvantage caused by racism. For a biosocial organization, such as RSCN, keen on fighting for the intersection of rights of a constituency that conceptualizes suffering in particular ways, it might make sense for its members to gatekeep based on the measures of authentic suffering. The merging of phenotypic and genetic identifiers used to speak about SCD creates a contested space in which the illness imaginary resides. Like the medical practitioners of the early twentieth century, Brazilian citizens who are phenotypically white link their disease status to an ancestor who was undoubtedly African. The historical pairing of SCD and Blackness takes real effort to dismantle. It is not the role of this paper to take this dismantling to task, but instead to offer an analysis of both the promise and peril of this linkage.

## Legitimate suffering and authentic belonging

In February 2014, two days before the beginning of Carnaval, I was scheduled to conduct an interview with Liza, the vice president of RSCN. When I arrived at the RSCN office, nestled in a corner on the eighth floor in HemoRio, I was told she was in a meeting. I probed further and learned that she was in fact participating in a meeting of regional SCD civil society leaders. I asked if I could sit in the back and observe and was granted permission. Coming in late, I went straight to the back but was soon urged to join the table. Seated at the table was the newly inaugurated president of Federação Nacional das Associações de Doença Falciforme or National Federation of Sickle Cell Disease Associations and representatives from all of the organizations based in Rio: RSCN, Mulheres com Doença Falciforme do Estado do Rio de Janeiro (MDF-RJ), Associação de Doença Falciforme (ADF-RJ), and two smaller organizations that represent both men and women, respectively, local to the city of Rio de Janeiro. I interrupted a roundtable in which fifteen people eventually participated—including local leaders Jorges; his wife, Anna; and Mira, the current president of RSCN. The leaders of each organization were relaying the needs and challenges to the new national president, who had made a trip to Rio from Belo Horizonte to meet with the director of the SCD Program for the Ministry of Health and other Rio-based practitioners of SCD. When it was Mira’s turn to speak as president of RSCN, she was animated and emotional—characteristics I was acquainted with by now when people spoke about SCD, especially in groups. It was the well-crafted performance of someone who was used to making appeals. She expounded on the successes of RSCN and fought for RSCN’s place at the national table and for her place as a leader, but the meeting eventually devolved into a heated confrontation between the leaders of the regional organizations. Angers flared, voices raised, and tears shed. This moment was about perceptions of power and legitimacy. Mira was the only one at the table who did not have SCD, and I strongly felt her need to justify her presence at the table.

Shared suffering via shared practices, experiences, and histories is the metaphorical adhesive for a collective. This conceptualization also allows us to understand that, for SCD, shared suffering takes place within the rare disease state realm as well as in the realm of social exclusion and disadvantage associated with Blackness. Brazilian identities in this study who have SCD have organized themselves into civil societies in attempts to make claims to the government, pharmaceutical companies, and the public health apparatus for the retrieval of financial support and material goods—but also to influence these entities for the betterment of their constituency. In this process, they have become important stakeholders who interact on every level with the development and implementation of policy. These patient activism groups are organized around a genetic mutation, yes, but they are also organized around a relatively recent racial consciousness and a recognition (made clear by the NHPBP) that racial discourses of suffering may be necessary to deploy to achieve their goals. These racial discourses of suffering are further complicated by the inclusion criteria made clear by those living with SCD—criteria set by measures of defined authenticity via indices of suffering.

The notion of authenticity, particularly among Afro-descendants and Indigenous people in Latin America, has been increasingly documented (Hanchard 1998; French 2009; Silberling 2003; Perry 2013; Forte 2013; Farfán-Santos , 2015). For Brazil in particular, the rise of affirmative action education and health-related policies has often brought into question who has the right to make claims to the benefits bestowed to certain citizens by the government based on race or color. In the case of land ownership, biological and cultural distinction is measured by African ancestry and direct linkage to African slaves brought to Brazil. In her empirical analysis of quilombos in Bahia, Farfán-Santos (2015, p. 112) argues that quilombo descendent communities’ authenticity “depends not only on their ability to perform and describe their ancestry and the history of their community, but more importantly in their ability to tell a specific history of their past as it has been written and incorporated into the Brazilian national imaginary.” She further contends, “the politics of cultural recognition depends on a ‘black culture’ that can be specifically defined and visibly identified” (Farfán-Santos 2015, p. 120). Here I discuss authenticity not of Black populations in the context of a larger Brazilian political system, but instead an authenticity that frames how those who have already negotiated space for themselves gatekeep entry into a group that has already established a foothold in how their political identity is managed. I argue that those who fit the inclusion criteria for an authentic experience of SCD will fail to legitimize those who do not meet the same criteria—even if the measures of authenticity are tangentially related. Legitimacy has been defined as “a general perception or assumption that the actions of an entity are appropriate within some socially constructed system of norms, values, beliefs, and definitions” (Suchman, 1995, p. 574). The literature on legitimacy and how it serves or disserves organizations and individuals has been well studied. Sonpar et al. (2009, p. 1) have suggested that “while an optimal level of legitimacy-seeking behaviors may be necessary for organizational effectiveness, an excessive focus on legitimacy may lead to stakeholder mismanagement and have the opposite effect…[and] that legitimacy may occur for reasons other than institutionalization of values and be temporary in nature.” In the case of organizational leadership for RSCN, if the modes of suffering are not mapped in a way recognizable to the gatekeeping biosocial group, fissures are created within that group.

During my time in Rio de Janeiro, I interacted with Mira at meetings and symposiums, in instances where she held the most power and also when she did not. As president of RSCN, she was on the front line to advocate for services and treatment of those living with SCD in Rio de Janeiro. Several identities emerged through Mira’s interview with me, her interactions with others, and general observations I made during my time with her: mother, leader, trait carrier, woman of affluence, and activist. At first glance, these identities may seem like appropriate ones to hold as the head of a reputable community organization, but there was one instance—the meeting described earlier—in which it was clear to me that these identities did not necessarily map onto the identities that align with those of her constituents.

During this meeting, conflict boiled over into an angry exchange of words between past and present leaders who have made up the civil society ecosystem for SCD in Rio de Janeiro. Near the end of the four-hour meeting, citing the need for democratic practices, one of the women stood and reprimanded everyone:

Do you know that while we're being so nitpicky for minimal things, do you know who's losing? The people who are losing are the people losing their lives…I am talking about the patients because those who are not patients, it is different; you can support the cause and be leading the cause, but you will never feel it in here [*hits her chest*]. You will never feel here what we feel. Our moms, our parents, they don't know what it's like, and will never know—because I know that my mom suffers, she would die next to me on my hospital bed. I don't doubt my mother's love, but to feel what we feel, she doesn't feel, she can't feel. I don't doubt that Mira has a good cause and that she fights with the suffering of losing her children, but feeling it here, knowing it here [*hits her chest again*], only we can know.

This woman’s monolog initiated a brief but powerful exchange that suggests that the suffering of a mother is not equitable with the suffering of those living with SCD. One might assume that this was said for the benefit of Mira, the only person around the table who had SCT and who identified strongly as a mother of children with SCD. In the context of the ongoing argument, there was a dispute seemingly between Mira as the representative of RSCN and Lucas as the representative of a men’s SCD group. Lucas, a known leader in the SCD world, is a dark-skinned male living with the homozygous variant of SCD (HbSS). Though it is never directly said, this woman appears to align herself with Lucas, and delegitimizes both Mira’s argument and her suffering.

I woke the next morning to an email from Mira sent to the officers of RSCN and me at 1:00 a.m. The subject heading read, “*Carta aberta de uma mãe de falcêmico*” (“Open letter from a mother of someone with SCD).” An excerpt of the letter reads:

What does the mother do when she sees her son is in acute pain crisis, screaming in pain and no medicine is effective?

What does the mother do when the child looks at her, after the crisis of pain, exhausted and with hopeless eyes? …

What does the mother do when the child dies in her arms at age 19?

SHE ALSO DIES!!!!

Who says that the mother with trait does not feel like the son, because she does not have the disease. It is to deny life…

What does the MOTHER do whose pain recurs with another child? **SHE DIES AGAIN!!!**

This MOM with her heart torn, puts together the small part of the heart that is left, because she has other children, and they also need this Mother and she **SURVIVES!**

**This Mother is me!**

**Shut up those mouths that open to say that a mother does not feel the same pain as their child.** (Mira’s capitalization and emphasis)

Mira’s letter speaks to the interconnected ways she makes sense of her place within the hierarchy of suffering: how her trait status puts her into contestation with understood suffering, her role as a mother to serve as a vehicle for legitimacy, and relatedly, an interpretation of (non-biological) pain as a mother of children with SCD*.* The letter also demonstrates her need for legitimization as a leader of a disease organization focused on SCD—a legitimization that also hinges on class and color. Though these are discrete categories, what you will find below in my analysis is that they are all interconnected.

### Trait status

Unlike the women highlighted in Fullwiley’s work (2006) who have SCT, Mira never claims pathology. The only pathology she recognizes are her children’s, but her trait status places her in between notions of normal and pathological. Fullwiley (2006) has discussed the suffering associated with SCT status in Senegal, usually embodied by women, and asserted by clinicians as being a psychosomatic problem. The carrier status, in this context, transmits a biosocial suffering. SCT, while not characteristically associated with the severe physical manifestations that come with SCD, is not completely benign (Mitchell 2018; Tsaras et al. 2009; Key and Derebail 2010). There have been reports of adverse conditions that occur due to a patient's trait status, and patients with SCT could have the same presentation as sickle cell anemia if they are exposed to conditions that favor sickling (Ashrobi et al. 2020). Further, SCT is often implicated in larger conversations about reproduction and risk. Very often, mothers who have SCT are unaware of their status and may have unknowingly contributed an S allele to their children diagnosed with SCD. In Hill’s (1994) study of low-income mothers with children diagnosed with SCD, she suggests that screening programs and education were possible to prevent the genetic transmission of SCD but were not embraced. Instead, structural barriers to healthcare and a cultural ideology of motherhood undermined the power of education to change minds. When education is not absorbed and subsequent reproductive choices to give birth are ignored despite the risk, and when these factors are mapped unto to women of lower socioeconomic status, this logic may explain Mira’s statement, via an interview with me, that had she known she had SCT, she would have never knowingly conceived: “Since my son had such a terrible case of SCD, I honestly say that if I’d known I had the trait, I wouldn’t have had a child. A lot of people want to kill me for saying that, but I wouldn’t put a sick child in this world…bringing a sick child into the world to suffer—I just couldn’t do it.”

Certainly, Mira’s organizational power struggles indicate that her inheritance of only one allele is problematic for others as she leads the organization. When I interview Mira, though she is vocal about her leadership, particularly the successes of that leadership, it is clear that she does not consider herself a person with SCD; nor does she want others to consider her a person with SCD. She never claims authenticity as delineated by others living with SCD. Her language is distancing, full of ‘they’ and ‘them,’ and steeped in ideologies of noblesse oblige. The deaths of her son and daughter fuel her fire and assist in her self-ascribed legitimacy. Mira wanted legitimacy from her constituents—we can sense that strongly in the email she sent to her members—but in order to be properly recognized, her constituents demanded an unspoken but understood authenticity from her. The letter she wrote is about the pain of having children with SCD, but focuses on her particular pain as a mother after the loss of those children.

### Motherhood

In a study about the activism of Latina and African-American mothers in the realm of education, Fuentes (2013) notes that many mothers come to their political awakening in organizing out a place of concern for their children or grandchildren, transforming their social identities as mothers into a political strategy. Political mothering describes collective mobilizations, such as the Mothers of the Plaza de Mayo, to challenge institutionalized injustice. The concept of ‘motherhood capital’ (Lo 2016) can help us understand the role of mothers in advocacy spaces and beyond, where women use their social roles as mothers to bargain for better outcomes for their children through individual negotiations. Motherhood capital involves individual strategies, rather than collective resistance. Mira clearly articulates how she was eventually called to action in memory of her son. At the core of motherhood capital is “the mother’s interactional styles and knowledge that signal to institutional gate-keepers their deep caring for and intimate understandings about their children” (Lo 2016; p. 695). In this case, the gatekeepers are not medical doctors in the healthcare system in which Mira must fight for proper treatment of their child. That time may have been apparent for her children when they lived, and Mira may have had to utilize this capital in that context, but, in present day, the gatekeepers were those who questioned her capability of properly fighting for RSCN members as their president. As defined by Lo (2016), motherhood capital is a non-elite cultural resource. Ironically, while Mira inhabits the high-status cultural signals (money, fair skin, connections) that allow her entry in other spaces, these same signals work against her in attempts to gain entry and acceptance with those affiliated with RSCN.

As explained earlier, the most frequent complication associated with SCD is pain. Pain precipitates more presentations for medical care than any other symptom. Pain is recognized as the predominant reason for presentations for medical care by adults with SCD (Edwards et al. 2005). In Mira’s short email response, she mentions the word *pain* five times and attempts to appeal to the reader to consider the pain of a mother who has had to live through multiple SCD-related deaths of her children as on par to the physiological pain associated with SCD. According to Alam (2012, p. 3), “mothers, in particular, traditionally have been expected to be the primary caregiver and play a direct role in the nurturing and development of their children, reflecting communal and caregiving traits fostered by societal expectations.” According to Brown et al. (2016, p. 2893), “caring for a person affected by sickle cell disease (SCD), can precipitate ‘chronic sorrow’ (Northington 2000)…due to its clinical variability (Ballas et al. 2010), treatment as a racialized disease (Bediako and Moffitt 2011; Rouse 2009), as well as financial stress and possibly daily disruptions in family interactions.” Mira’s email is trying to make the reader understand her spectrum of pain—pain encountered through birth, pain akin to SCD endured at diagnosis that lasted until death, and an enduring pain that she must live through as a result of those deaths. She is also attempting to provide the reader with the righteous justification of her leadership and long-standing service to others.

In one of our interviews, I asked Mira about where she came from. She spoke about her childhood in Laguna, Santa Catarina, in Southern Brazil, her father’s abandonment of her and her siblings, and the respect her grandmother evoked.

Mira: I had lots of boyfriends. When I got married, it was basically because my grandmother forced me to get married to see if I’d give less trouble for the family. But I liked him—I got married to a guy I liked. I wouldn’t have gotten married otherwise. At that point, funny, I was already involved in politics…Then I went to college. I met my husband there, we got married, and I had my four children. They have all graduated. I lost two and I have two.

MC: Lost two to SCD?

Mira: Well, yes, I didn’t know my son had it, even though he always showed the characteristic symptoms. Now I know what they are, but I didn’t know. When he died, he was already in his second year of engineering at UFRJ. He was really smart. When he died, he had a really, really bad pain crisis and he turned all yellow. He died in my arms. That day, we did the tests at the Hospital of Santa Cruz and they said, “No, this is not rheumatic fever. This boy has SCD.”

Mira did not get involved with the SCD movement immediately after her son’s death. It was not until 2008 that she brought her skills to RSCN. She explained,

I forgot about SCD. I didn’t want to hear about it. I became a leader for protecting the rights of disabled people. So, I had a lot of positions, working during a number of governmental administrations. I worked with the governor and always for rights of disabled people…Then I met Marcos, who was the ex-president of RSCN. The governor had placed me as the representative for the palace at the Council for Disabled People…and one day at a meeting Marcos had a crisis and that brought everything back for me. Everything I’d tried to forget, drown out, came flooding back. So, I said to him that he’d made me remember my son. He said, “You have to come to our meetings.” I said, “No, I don’t want to. I have already suffered so much.” But he insisted so much that I came. So, I started to participate. I’m such an intense person that when I get involved with something, I do it with all my energy. And I dived headfirst into this struggle.

The practice of ‘memorialisation’ (Gibbon and Novas 2008, p. 21) is also described as a way that breast cancer civil societies identify themselves via the remembrance of women who have had or have died with the condition. Mira takes on an individualistic version of memorialization through her work with RSCN on behalf of her children. She occupies space on two planes: one in which she is connected biologically (even if by only one allele) to SCD, and one in which her normal hemoglobin outweighs any perceived ‘value’ as distributed by those who have two alleles. Neither the sickle allele nor the normal one allows her clean entry into a biosocial grouping. To Mira, however, her allele contributed to the disease status of her children and, as a result, should firmly situate her among everyone else. Her genetic contribution to the death of her children authenticates her placement in the biosocial group of those with HbSS, even if the group does not authenticate her.

One might imagine that as a mother, especially one of two deceased children, Mira might feel as though she is more affected than if she had SCD herself (Almeida et al. 2006; Burnes et al. 2008). We might also imagine that her suffering is a product of her own and unwilling ignorance produced by a system not yet adept in knowledge about SCD. Hill (1994, p. ix) posits that the meanings these women “construct and assign to the SCD experience develop from their own values, resources, and life experiences”—even if the knowledge of a SCD diagnosis is delivered retroactively. Burnes et al. (2008) conducted a study in Canada to assess the experience of mothers raising a child with SCD and found great frustration about the lack of knowledge and skills from providers surrounding the treatment and care of SCD. Yet this angst is not enough for some of the members of RSCN, and there are those who demand ‘authentic’ representation. This authenticity, for many, is delivered via the homozygous form of the disease.

### Color and class

As we have seen, Blackness is also an authenticating identity that is understood to be associated with SCD. Notably, around the table that afternoon, Mira was not only the sole person in the room with SCT, but she had the lightest skin color in the room. Could her lack of authentic representation be attributed to not only genotype but phenotype as well? Could the absence of Blackness be a contributing factor to the discomfort some people feel in her leadership? Mira self-describes as *mulata* and mentions that her grandmothers are *Negra* (Black), her mother is *Branca* (white), and her father is from the northeast. Though she never mentions his race, I assume, based on all the other familial descriptors, that her father is *Negro* (non-white). Due to the close association that Blackness has with SCD in Brazil, arguments about the authenticity of her phenotype are likely embedded in the arguments that question her genetic status as person who can relate and thus lead a SCD organization. In fact, her societal display of whiteness is what she attributes to the lack of diagnosis for her children. No one suspected that she or her white husband could carry the trait. This notion of invisibility is potentially transplanted through Mira as leader and, in many eyes, threatens the credibility of the SCD organization.

Could her phenotypic lack of Blackness also provide what Dressler and Bindon (2000) define as a cultural mode of lifestyle that is non-relatable to most of her constituents? Lifestyle in this sense is defined “as the accumulation of consumer goods and the adoption of behaviors that help to define one's social identity” (Dressler and Balieiro 1996, p. 331). Throughout my interview with Mira, she discusses points of privilege that set her apart, at least class-wise, from others I interviewed associated with RSCN. Mira has attended college, her children have all attended university, her husband is a lawyer, and on entering the SCD movement, she remarks, “I abandoned all my other jobs; I had the freedom to do that because I didn’t really need the money.” On the other hand, Mira’s phenotypic spectrum may lend credibility to those outside of the civil society. Despite this, I suspect, many other evidences of turmoil, Mira is currently still the president of RSCN and has been for eleven years now. Even if she is not recognized biologically and culturally as having earned full entry into the biosocial space, the very identities that repel that entry may be tolerated for the operationalization of her leadership role.

Putnam (1995, p. 611) suggests a link between collective identity seen in social movements and social capital in his statement that “a grassroots political movement…is a social capital-intensive form of political participation.” High levels of social capital are required for political effectiveness. In the case of the SCD movement, a movement that constitutes individuals who decry their neglect and attribute that neglect to racism, discrimination, and lack of access to social capital, Mira’s presence (wanted or not) may offset some of the group’s low level of social capital. In her study on the issues and strategies of advocacy groups who serve racial minorities, low socioeconomic populations, and women, Strolovitch (2008, p. 24) notes that advocacy groups can be disenfranchised along several axes: “they might lack financial resources; they might now be or have been in the past the objects of de jure facto discrimination; they might lack electoral power and therefore have no or few elected representatives; or they might lack ‘cultural capital’ because they a socially stigmatized by the broader society or dominant culture.” Mira likely capitalizes on the cultural markers of whiteness (high education, high-skilled employment, high income) that allow for learned savvy in institutional spaces. Simultaneously, these same markers negate any suffering that are mapped onto the typical lived experience of those with SCD.

## Conclusion

What I have attempted to show is that the biosocial nature of patient activism organizations, though often cohesive around shared objectives, may not be align on shared experiences of suffering. The implications of this are significant when the ‘face’ of the organization, the presumptive leader, is excluded in different ways by the very constituency she serves. While embodying identities that allow for social privilege in the Brazilian society, a space where people living with SCD are often excluded, those same identities give her limited access to shared understanding of suffering. In short, does not map unto either the physical pain typically associated with SCD nor the pain and suffering attached to norms of disadvantage that are embodied in Black bodies. In Brazil, the authentic experience of SCD is performed in a particular body. It is a body in which inequality has been embodied as a result of racism and other forms of historical oppression, usually most severely in those who are dark-skinned. The suffering that is simultaneously embodied by the compounding effects of racism within a body already experiencing debilitating pain creates shared histories, experiences, narratives of hardship, and resilience. Gatekeeping aligns itself along particular axes of suffering. In the case of Mira, no one disputes the pain that she must have endured by living through the deaths of her children with SCD, but that pain and level of suffering is not seen as equitable. For this woman living with SCT and the memories of pain as ascribed unto her children, it is still not enough for full inclusion.

Yet, in her role as president, she is not the product of full exclusion either. Her identities of fair-skinned, middle-class, and singular allele carrier are in direct opposition to not only how SCD has been constructed in Brazil, but also to her subordinates and organization constituents. The result of this misalignment may result in the discursive resistance to her leadership as evidenced in the meeting I witnessed. Outward facing, that resistance is not as evident. Mira’s continued and current leadership suggests that there is a spectrum of legitimacy that she is being offered here. While perhaps never deemed authentic to the subordinates within the organization, her connection to suffering allows for her to be heard and to claim space as a leader. Even as the aughts brought varied success in the form of judicialization to the activism of SCD, it is still important to note that comparably, when it comes to the advancement of care, research and development of new therapeutics, or even increased health promotion and education, SCD continues to lag behind other disease states. Mota et al. (2017, p. 287) note approximately eight years after the National Policy for the Black Health Population was implemented, that “the policy neglect of SCD, could be attributed to institutionalized racism, in which the needs of ethnic minority groups [are] ignored or misrepresented.”

As stated earlier, I am interested in how suffering may help us interpret authenticity claims, the kinds of suffering that are allowable and acceptable for inclusion into SCD patient activism spaces. How might hierarchies of suffering help explain complex modes of gatekeeping, particularly when institutional gatekeeping in the broader societal context is the norm for those who are wielding the power in an organizational microcosm? These microcosmic communities are also performative—as made clear by the internal and external machinations of RSCN. In my larger project, I found that the spectrum of skin color correlates with the real and perceived benefits of formal citizenry. Those actors living with SCD who are on the lighter end of the color spectrum tend to be afforded more rights and have less need to call on and demand their rights in biological terms. Many of the dark-skinned Afro-Brazilian respondents in that study lived lives as second-class citizens, and their participation in civil society (specifically the *Movimento Negro* and the SCD movement) was a way to fight for more rights. In the narratives I collected, the more hardship my respondents endured, the more they were inspired to join a collective entity to aid in their sense of belonging. The subordinates of RSCN who are legitimized by the state as being deserving of policy, care, and treatment as a result of suffering attached to both social and biological catalysts may feel resentful to those who may not need to rely on the services that RSCN helps supply. The fluidity expressed in so many aspects of Brazilian culture may also allow for the fluidity in which Mira is both accepted and rejected.

The biosocial underpinnings of suffering offer us a way to conceptualize the suffering and assumed recourse of judicialization that will be associated with the global pandemic of COVID-19 in Brazil. How might identities within these movements be contested? How might claims of suffering be doubly neglected along both biological and biographical lines with the current right-wing government? Could the stripping away of the rights so hardly fought for once the Brazilian government began to open up after the last dictatorship, further make malleable the hierarchies of suffering? Perhaps in deeper scarcity, different identities will hold value differently and modes of exclusion will transform.
